# An Instance Segmentation and Clustering Model for Energy Audit Assessments in Built Environments: A Multi-Stage Approach

**DOI:** 10.3390/s21134375

**Published:** 2021-06-26

**Authors:** Youness Arjoune, Sai Peri, Niroop Sugunaraj, Avhishek Biswas, Debanjan Sadhukhan, Prakash Ranganathan

**Affiliations:** School of Electrical Engineering and Computer Science (SEECS), University of North Dakota (UND), Grand Forks, ND 58201, USA; youness.arjoune@und.edu (Y.A.); sai.peri@und.edu (S.P.); avhishek.biswas@und.edu (A.B.); debanjan.sadhukhan1987@gmail.com (D.S.); prakash.ranganathan@und.edu (P.R.)

**Keywords:** clustering, heat loss quantification, instance segmentation, Mask R-CNN, deep learning, mean average precision, thermal imagery, UASs, U-value, ASHRAE

## Abstract

Heat loss quantification (HLQ) is an essential step in improving a building’s thermal performance and optimizing its energy usage. While this problem is well-studied in the literature, most of the existing studies are either qualitative or minimally driven quantitative studies that rely on localized building envelope points and are, thus, not suitable for automated solutions in energy audit applications. This research work is an attempt to fill this gap of knowledge by utilizing intensive thermal data (on the order of 100,000 plus images) and constitutes a relatively new area of analysis in energy audit applications. Specifically, we demonstrate a novel process using deep-learning methods to segment more than 100,000 thermal images collected from an unmanned aerial system (UAS). To quantify the heat loss for a building envelope, multiple stages of computations need to be performed: object detection (using Mask-RCNN/Faster R-CNN), estimating the surface temperature (using two clustering methods), and finally calculating the overall heat transfer coefficient (e.g., the U-value). The proposed model was applied to eleven academic campuses across the state of North Dakota. The preliminary findings indicate that Mask R-CNN outperformed other instance segmentation models with an mIOU of 73% for facades, 55% for windows, 67% for roofs, 24% for doors, and 11% for HVACs. Two clustering methods, namely K-means and threshold-based clustering (TBC), were deployed to estimate surface temperatures with TBC providing consistent estimates across all times of the day over K-means. Our analysis demonstrated that thermal efficiency not only depended on the accurate acquisition of thermal images but also relied on other factors, such as the building geometry and seasonal weather parameters, such as the outside/inside building temperatures, wind, time of day, and indoor heating/cooling conditions. Finally, the resultant U-values of various building envelopes were compared with recommendations from the American Society of Heating, Refrigerating, and Air-conditioning Engineers (ASHRAE) building standards.

## 1. Introduction

Heat loss quantification (HLQ) plays a crucial role in reducing the overall energy consumption for optimal operations in buildings, particularly since its usage has a considerable impact on the environment and a building’s life cycle [1,2]. A key objective of HLQ is retrofitting existing building envelopes. The first necessary step in the building envelope optimization process is assessing the actual thermal performance. Indicators, such as the energy performance or energy use intensity, are used to express this performance.

The building envelope in-situ measurement depends on factors that are classified into three main categories: site conditions, building conditions, and operating conditions [3]. The site conditions category refers to the weather conditions under which the tests are performed. These weather conditions include, but are not limited to, wind velocity, rain, solar radiation, and humidity, all of which can significantly alter the building’s thermal performance quantification.

The building condition category refers to the age of the building materials and the laying of the structural elements used during construction. The operating conditions category refers to the building’s environmental management, such as heating or cooling, and air circulation from the opening or closing of windows, and building maintenance, regardless of whether or not these activities are currently affecting the building envelope. All of these factors must be monitored and considered carefully during the evaluation of building heat loss quantification.

Multiple research groups have recently investigated the use of infrared thermography to measure building envelope parameters in-situ with the thermal transmittance values, or the amount of heat-flow in one square meter when the temperature difference is one Kelvin (U-value). Most of these techniques present unique challenges [4]. The in-situ-based measurement of the U-value, along with the heat flowmeter method (HFM), is not always possible [5] or accurate [6] due to the assumptions upon which the HFM methods are based, such as uni-directional heat flow; therefore, it is of crucial importance to develop practical techniques that quantify the heat loss.

One technique to obtain thermal imagery is by using drones, which creates opportunities in building assessment and inspections [7,8,9]. Drones enables fast and safe building inspections, which are necessary to complete proactive maintenance to mitigate problems before they become costly. Reducing the costs associated with insurance inspections is another benefit to building owners and managers. The risks associated with using drones for roof inspection are low compared to traditional methods, where employees risk injury as they traverse the building to inspect the structure. Drones are increasingly used for data collection; however, thermal images captured by drones often contain objects, such as trees and ground surfaces, all of which can impact the calculation of the U-value calculation [10]. Instance segmentation of the regions of interest, such as the facade of the building or roofs, is a necessary step after collecting data.

Over the past two decades, several instance segmentation and masking techniques have been considered using machine learning-based methods, which are the most successful at identifying objects [11,12]. Machine learning applies complex mathematical models to uncover hidden correlations between the different features in a given data set. There are two types of machine learning techniques: supervised and unsupervised. Supervised techniques require specific rules that an expert provides for the machine. These rules allow the machine to either classify or predict the outputs of the model given an input. Unsupervised techniques are applied when an expert cannot provide rules because of the large data sets. These techniques are applied to reveal any hidden correlations that the expert may not notice. Machine learning techniques require feature selection, which requires human expertise to determine appropriate features. A recent class of machine learning, called deep learning, does not require this step and is a new and powerful technique for computer vision tasks, which has not fully exploited by the heat loss research community [13,14].

In addition, the current research in heat loss quantification has relied largely on qualitative bench-top solutions or localized analysis in energy audit building evaluation. The existing quantitative models used to estimate surface temperature in buildings do not account for multi-variate uncertainties, such as energy requirement/consumption patterns, multiple sets of images per building object, time-of-day, seasons, and building material. There are also inconsistencies in the process of arriving at a reliable and quantifiable U-values for a building envelope.

For example, the thermal readings taken from a combination of thermocouple sensors and a thermal camera are only “raw values” that need to be pre-processed and subjected to multiple uncertainties (e.g., solar radiance, wind speed, time of the day, and black body radiation) and, thus, require the need for post-processing stages. In addition, the current literature does not provide guidance on how to arrive at an optimal and accurate way to detect building objects through which one can estimate surface temperatures from the region of interest (ROI) through heat loss metrics. Existing solutions are, therefore, not reliable for energy audit applications.

The specific research questions our work addresses include:

1. How do we acquire and process thermal images that account for building geometry uncertainties, such as orientation and angle, seasonal changes, and  the influence of weather parameters on the building envelope?
2. How do we accurately detect various instances of objects, such as walls, roofs, and windows, using data-driven approaches?
3. How do we automatically tag or label images and report them?

Our work covers a comprehensive data-driven approach that examined approximately 100,000 thermal images, and performed object identification to classify objects accurately using instance segmentation to detect various building envelope structures, such roofs, walls, doors, windows, and facades. We also created a method for automated tagging, tracked pixel–pixel surface temperature values and reported the values in quantifiable and standard U-value estimation units.

There exists hundreds of variations of U-value formulae in the existing literature, and often they do not use large data but instead single point values. We investigated all available U-value equations and developed a cumulative U-value formula from three existing U-value equations. The U-value is a series of heat transfer equations that account for pixel temperature, outside temperature, wind speed, etc., to develop a quantitative measure of how that particular object is performing compared to how it should theoretically be performing. It can be thought of as an extension to the direct thermal readings, but we account for it from multiple low ceiling thermal images per building object acquired from small scale aerial systems.

We deployed two clustering methods (e.g., K-means and threshold-based clustering), which were then developed to estimate the accurate surface temperatures of multiple instances of an object. Then, using the estimated surface temperature for the region or envelope, we developed a cumulative U-value (*U*${}_{c}$) formula that uses multiple existing U-value equations from the literature. We empirically verified our *U*${}_{c}$ as the most accurate formula when using a benchmark to meet the ASHRAE standard recommendations. *U*${}_{c}$ demonstrated relatively lower errors compared to the other U-value equations. The statistical difference of the U-value building envelope computations against ASHRAE varied between 0 to 30% depending on area size, building type, and material used. Since our AI model can detect multiple instances of any object with greater accuracy, including the roof, windows, doors, HVACs, and facades, the model is unique, it fills the research gap of inaccuracies and provides a quantifiable way to address uncertainties.

Our work adds to the body of knowledge by addressing the lack of automated solutions in energy audit applications and providing a comprehensive view of the building envelopes that will result in reliable, quantifiable, and scalable workflows to address heat loss quantification problems for next-generation building inspection problems. We determined that the thermal efficiency of a building depends on multiple factors, not only on the accurate acquisition of thermal images, but on factors such as the building geometry, season of the year, time of day, indoor heating or cooling conditions, past historical consumption, and power generation sources. These factors are all influential in determining the overall assessment of an energy audit evaluation.

In this paper, we demonstrated that thermal imagery to quantify heat loss combined with the recent advances in deep learning theory has many advantages, such as remote sensing, flexibility, and minimizing injury risks. To the best of our knowledge, the proposed approach is the first of its kind. U-values for different building blocks were analyzed and compared to the American Society of Heating, Refrigerating, and Air-conditioning Engineers (ASHRAE) building standards. This approach will allow stakeholders to overcome the challenges of traditional heat loss quantification methods. This work expounds upon previous publications [4,15] with the following new subject material,

- A large thermal image data repository (∼100,000 images) of multiple university buildings was collected using a UAS and manually annotated to highlight objects of interest, such as facades, walls, trees, roofs, and windows.
- Multiple models for object detection, such as Mask R-CNN, Fast R-CNN, and Faster R-CNN, were trained on several backbone types and validated with metrics, such as average precision (AP) and intersection-over-union (IoU), through a data-driven three-layered framework.
- Two clustering schemes were tested to estimate surface temperature readings and identify hotspot regions reliably. Quantified surface temperature observations were used to compute the U-values of objects and validated with the ASHRAE standards.

The relationship between the indoors and HLQ is essential; however, this is out of the scope of this paper since our main focus is to provide a heat loss estimation using thermal imagery of the buildings from the outside and from which the heat loss is determined.

The rest of this paper is organized as follows: Section 2 presents state-of-the-art techniques for object detection and instance segmentation. We devoted a specific section for this and did not integrate it as subsection of the introduction so as to not interrupt the flow of the paper as this section provides an in-depth review of the recent advances in deep learning and computer vision. This section provides also useful knowledge that can help in developing novel computer vision techniques. Section 3 describes the methodology used in this paper to quantify the heat loss. We describe the training and testing methodology of several computer vision techniques as well as the analytical formulas used to calculate U-values through a three-layered framework. Section 3 also describes the clustering techniques applied to detect hotspot regions within thermal images. Section 4 presents the evaluation metrics and examples of the obtained results, which includes the results of instance segmentation, clustering analyses, heat loss quantification using U-values, and a qualitative and quantitative uncertainty analysis. Section 5 summarizes the paper, presents our conclusions, and suggests future research work.

## 2. Related Work on Computer Vision and Image Clustering

In this section, we provide an overview of the recent advances in computer vision and the state-of-the-art algorithms for object detection and segmentation. This section provides also some related work on image clustering and the detection of hotspots.

### 2.1. Object Detection and Instance Segmentation: The State of the Art

Computer vision can be classified into either object detection or segmentation. Object detection is more specific than classification in that it must draw a bounding box (BB) around every object identified [16]. If an object detected has been identified completely, including all pixels, it is considered a segmentation. Segmentation methods can be further divided into semantic, instance, and panoptic. All pixels belonging to all objects of the same class are classified as one image segment in semantic segmentation. Instance segmentation classifies each instance as a segment even if the image is formed of objects of the same class.

Panoptic segmentation combines both instance segmentation and semantic segmentation by assigning class labels to each unique object segmentation. Object detection and segmentation has been performed in the past by using traditional techniques, such as histogram gradients. Deep learning in computer vision is gaining popularity, as it has been recognized as an effective technique compared to traditional methods [17]. Table 1 summarizes the popular machine learning models used to perform computer vision tasks, with a brief discussion on their performances.

Applying a classifier, such as a convolutional neural network (CNN), for detecting the presence of an object within each region of interest by splitting the images is an incomplete approach for deep learning. CNN, concatenated with a classifier, such as fully-connected layers (FCs), cannot be used for the number of object occurrences in each image since the objects are not the same and, thereby, change the length of the output layer. Region-based CNN (R-CNN) has been proposed to mitigate this issue. This algorithm extracts region proposals with approximately 2000 regions using a selective search algorithm.

These proposals are then used to form warped regions on which a CNN is applied for feature extraction. This latter feature extraction is fed to a support vector machine classifier to classify the regions. Even though R-CNN performs well, it must repeat this process for all images, each of which requires 2000 processed regions. Each test image requires 47 s to process; therefore, R-CNN is infeasible even though it performs well. R-CNN achieves an mAP of 62% on PASCAL-VOC-2012.

A method utilizing a similar approach to R-CNN with some manipulations was proposed to mitigate the time constraints issue of R-CNN: Fast R-CNN [20]. Fast-R-CNN generates feature maps from the input images using CNN instead of feeding the region proposals to CNN. The region proposals are then identified and processed into squares. Regions of interest pooling is then applied to reshape the warped regions into a predetermined size, forming the input for an FC. The output layer of the FCs consists of a SoftMax classifier alongside a bounding box regressor. Fast R-CNN only required 0.32 s for testing and 8.75 h for training. R-CNN required 47 s for testing and 84 h for training. Fast R-CNN achieved an mAP = 39.3@0.5 on MS COCO dataset with 2000 region proposals and 66% on PASCAL-VOC-2012 [20]. Fast R-CNN trained VGG16 networks nine × faster than R-CNN and was 213 × faster at test-time.

Fast RCNN and R-CNN both use selective search algorithms to determine the regions of interest (ROI); however, the processing time is a limitation for both methods. Shaoqing Ren et al. [21] proposed an object detection algorithm similar to Fast RCNN, called Faster R-CNN, to overcome this limitation. This algorithm consists of a separate network to predict the region proposals, eliminating the selective search algorithms. Faster R-CNN with a ResNet101 backbone and FPN to extract the feature maps achieved an mAP=42.7% with 300 regions when tested on the MS-COCO dataset and 78.8% mAP on the PASCAL-VOC-2007 test set.

The previous regions-based detection algorithms perform predictions multiple times for various regions within each single image, which is a time-consuming task; therefore, You Only Look Once (YOLO) has been proposed [24]. YOLO models the detection task as a regression problem instead of using a region proposal. Each image is divided into several grids with two defined bounding boxes, increasing the speed of the detection algorithm. For instance, YOLOv3-320 processed images in real-time at 45 frames per second using Darknet-53 as a backbone, achieving an mAP=28.2%, while YOLOv3-416 achieved an mAP=31%, and YOLOv3-608 achieved an mAP=33%. YOLO can achieve real-time object detection; however, it has several limitations, such as the loss function, which treats the errors induced by small and large bounding boxes equally.

The authors of [27] created the first Fully Convolutional Network (FCN) trained end-to-end for image segmentation used in semantic segmentation. Many variants of FCN have been proposed, such as Graph-FCN, which achieved an mIOU=65.91% on Pascal-VOC Dataset and an mIOU=36.64% with FCN-32s on a PASCAL-Context dataset

The authors of [28] proposed an improvement of the model FCN, called ParseNet. This model improves upon FCN by allowing for global context inclusion in semantic segmentation. Relying on the largest receptive field of the FCN network is not sufficient for providing global context, and the largest empirical receptive field is not sufficient for global capture. ParseNet Baseline and ParseNet trained on the VOC2012 test set achieved a 67.3% and 69.8% mIOU, respectively.

The authors of [26] developed DeconvNet, a convolutional neural network (VGG-16) concatenated with a deconvolutional neural network (DNN) for semantic segmentation. The CNN-VGG-16 consists of the pooling needed to generate feature maps from the region in which the proposals are fed, which are then fed to the DNN. The DNN then performs the unpooling to determine the pixel-wise probabilities belonging to each class. The model was evaluated on PASCAL-VOC-2012 and was compared to the state-of-art segmentation algorithms. This model achieved a mean average precision of 69.6%, and some of its variants achieved a mean average precision of around 70%.

The authors of [34] proposed U-Net, a convolutional network for image segmentation, which is built on FCN. U-Net is composed of two paths or two sides: contractive and expansive. The contractive side has an FCN-like architecture extracting feature maps, while the expansive path spatially localizes patterns in the image subject to segmentation. U-Net was the winner of the of the EM segmentation challenge in 2015 and also the ISBI cell tracking challenge of 2015, with an IoU of 0.9203 for the “PhC-U373” dataset and an IoU of 0.7756 for the “DIC-HeLa” dataset.

The authors of [35] proposed DeepLabv3: which improved DeepLab by combining the parallel and cascade modules found within the atrous convolutions. The ResNet architecture was modified to maintain higher resolution feature maps within the same convolution.

Mask R-CNN can efficiently detect objects while simultaneously generating a high-quality segmentation mask for each instance [36]. A CNN network was added to the model parallel to the object detection task to determine the mask or the pixels belonging to the objects. Mask-RCNN does not support real-time analysis and is made up of two blocks. The first block, or backbone, deals with generating region proposals, while the second block, the ROI classifier and Bounding Box Regressor, classifies the regions proposals and generates the bounding boxes and masks.

The backbone consists of a standard convolutional network, typically ResNet50 or RestNet101, which serves as a feature extractor. As the features passing through the backbone network, the images are converted from 1024×1024×3 (RGB) to a feature map of shape of 32×32×2048. The new feature map serves as the input for the second block.

Mask R-CNN uses a Feature Pyramid Network (FPN) as an extension that can improve the standard feature extraction. FPN enables access to both lower and higher-level features. The Region Proposal Network (RPN), a type of lightweight neural network, scans over the backbone feature map once it is generated. The regions over which the RPN scans are performed are called anchors; for each anchor, the RPN generates an anchor class consisting of either a foreground class or a background class. The foreground class identifies whether or not there is an object in that box. The background class is the Bounding Box Refinement, which is a foreground anchor. This foreground might not be centered perfectly over the object, and thus, to refine the anchor box, the RPN estimates a change in the box’s coordinates, also referred to as delta (Δ).

The second block of Mask R-CNN runs on the regions of interest proposed by the RPN. The bounding box refinement step in the RPN causes different sizes for the ROI boxes that must be adjusted to the same size; therefore, ROI Align, a new feature of Mask R-CNN, is used to create a fixed input for the ROI classifier. The stride is not quantized in ROI align, and bi-linear interpolation is considered, while Faster R-CNN uses a quantized stride. The RPN classifier generates two outputs for each ROI: the specific class of the ROI object and the bounding box refinement.

**Figure 1:**
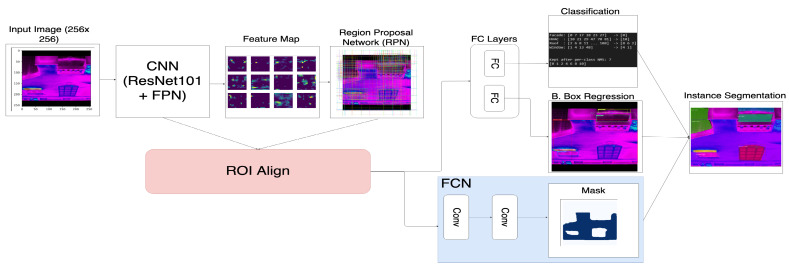
Illustration of the Mask R-CNN architecture with an input image at each stage of detection.

The bounding box refinement works further to refine the location and size of the box to encapsulate the ROI object. The last step of Mask R-CNN is the generation of segmentation masks. The segmentation mask branch consists of a convolutional network, which utilizes the positive regions selected by the ROI classifier and generates a mask. The full architecture along with the output at each step, is illustrated in Figure 1.

The authors of [30] developed a model, called the Context Encoding Network (EncNet) for instance segmentation. This model is built upon of two building blocks: the first of which consists of a CNN with different backbones, also called ResNet, to generate the feature maps. The output of this last CNN layer is fed into the second block, which is a context encoding module. The Context Encoding Module’s outputs are then reshaped and processed by a dilated convolution strategy while simultaneously minimizing binary cross-entropy losses and a final pixel-wise loss. The proposed EncNet with Resnet101 achieved an mIOU of 52.6% on the PASCAL-Context dataset.

The authors of [33] proposed panoptic segmentation to unify semantic segmentation and instance segmentation. To accomplish panoptic segmentation, the authors proposed a new quality metric to evaluate the overall segmentation. The evaluation metric can then be written as the product of two terms: the segmentation quality (SQ) and one recognition quality (RQ). Machine panoptic segmentation on instance segmentation with Mask RCNN + COCO achieved $PQ^{Th}=54.0$, 79.4, 67.8 on the Cityscape dataset, and the PSPNet multi-scale achieved a $PQ^{St}=66.6$, $SQ^{St}=82.2$ and $RQ^{St}=79.3$.

### 2.2. Image Clustering: The State of the Art

Image segmentation techniques have been used to identify, classify, and process regions of interest within colored and red-green-blue (RGB) images and, more recently, within infrared (IR) images. Table 2 provides a brief summary of image clustering methods and their performances.

The authors in [37] used a hybrid approach in their 2016 segmentation paper, that deploys the K-means and the Density Based Spatial Clustering of Application with Noise (DBSCAN) segmentation approaches to identify ’hotspot’ regions within IR images of photo voltaic (PV) arrays. Image pixel color values were first normalized and then pre-processed using the K-means method to segment the image into discrete regions of colors. This method creates distinct silhouettes of the various color profiles within the image. DBSCAN was then applied to obtain the pixel regions, which are above a set threshold of saturation in the hue–saturation–value (HSV) color palette.

Hajela et al. [38] used a 2D spatio-temporal analysis to detect and cluster regions of crimes, which were identified as hotspots. K-means was the primary approach used to classify different regions within a dataset that contained (x,y) co-ordinates, times, and dates for the events in each image. The number of instances of these K-means clusters were calculated and passed through a threshold to discretely obtain regions of hotspots within an image. This threshold was set based on the number of instances of each cluster and the total number of clusters. When combined with ensemble machine learning models, the use of clustering indicated a marked increase in the accuracy of crime prediction across various crime categories, such as vandalism, bribery, and extortion.

Another dual clustering method was introduced by Tamilkodi and colleagues [39], where the authors utilized a two-part process to cluster pixels within an image. The RGB query image was pre-processed to gray-scale. A histogram analysis performed based on the intensity or the brightness values of the gray scale image followed. This histogram serves as a one-dimensional space for a K-means based approach to cluster pixels; however, the novelty in this approach is the calculation of two-dimensional gradient with vectors that to point to higher intensity value pixels. This approach also processes these pixels as black or white based on a threshold ‘H’. This method was tested on a set of 1000 images from the Signal and Image Processing Institute (SIPI) and divided into ten groups based on similar content, such as dinosaurs, houses, oceans, horses, and others. Outcomes of 80% and 58.3% for the average precision and recall metrics, respectively, were produced by this method.

The authors in [40] utilized a clustering method based on Intuitionistic Fuzzy Set (IFS) theory and Fuzzy C-means (FCM) to segment images generated with magnetic resonance imaging (MRI). The C-means algorithm does not perform well with noise; therefore, the Intuitionistic Fuzzy C-means with Spatial Neighborhood Information (IFCMSNI) method proposed by the authors was used to preserve valuable spatial information through a ’spatial neighborhood information’ equation. The outcomes of this method were tested with a gray image MRI dataset with varying levels of noise through metrics, such as the dice score (DS) and the average segmentation accuracy (ASA), which was provided with ground truth data. These metrics indicated a significant improvement over existing methods, such as the Modified Intutionistic Fuzzy C-means (MIFCM) and Fuzzy Local Information C-means (FLICM), in the presence of Rician noise.

The authors in [42] introduced DEMP-k (Directly Estimated Misclassification Probabilities), which is a combination of the HoSC-K-means (Homoscedastic Spherical Components) and hierarchical linkage functions, thereby increasing the speed and performance of the algorithm. Their work proposed a framework for hierarchical merging based on pairwise overlap between components, this was further applied to the K-means algorithm. The model produced the results in Table 2 when tested on a digit recognition dataset.

A novel approach, called the Iterative Partitioning-Mean Shift (IP-MS) was introduced by Naik and colleagues [41], where the number of centroids chosen for each cluster and the number of iterations are key parameters for image segmentation. The image was pre-processed by reducing the noise, transforming the RGB image to a LAB color space, and normalizing the pixel values. The clustering algorithms then classified each pixel by finding the minimum Euclidean distance between pixels for each centroid and calculating the mean distance value for each cluster. Once the mean equals the number of centroids specified by the algorithm, convergence is reached, or the algorithm has successfully executed. The results of this algorithm indicated a marked performance increase in the accuracy and computation time when compared to the K-means algorithm.

## 3. Methodology

In this section, the methodology for data preparation, preprocessing, and evaluation is described. First, we start by describing the data-driven three layered framework to provide the complete picture of the process, then we discuss the building block data preparation. Second, we present K-means and Threshold-Based Clustering for hotspot detection. Last, we describe the U-value analysis using four formulae.

### 3.1. Data-Driven Three-Layered Framework

**Figure 2:**
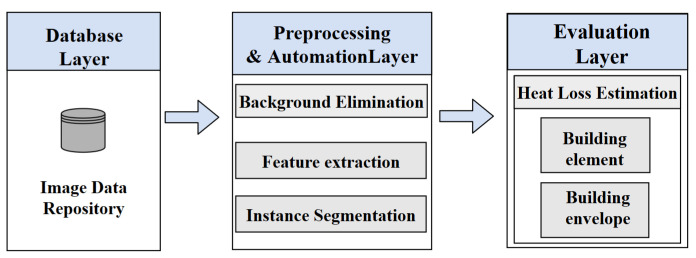
Data-driven approach for thermal performance [4].

Infrared thermal imagery is promising due to its extensive features, high performance abilities, and relatively lower cost. The thermal images must be pre-processed and automated before any meaningful information is collected. The image pre-processing includes the removal of unwanted background objects and the detection of inspected elements, such as windows, doors, walls, and other features. The current published research that addresses the terms of automating the methods for background removal, object detection, and U-value estimation is limited [43,44,45]. We, therefore, propose a fully automated three-layer framework for the U-value estimation of a building and its elements. Figure 2 illustrates a data-driven approach for the thermal performance assessment of building envelopes.

The raw thermal imagery captured from various sources, such as aerial or ground measurements, is stored in a data repository or database layer. The images are fed into a pre-processing and automation layer, where a series of background elimination steps are completed, and the critical features from the thermal images are extracted (refer Figure 2). The different building elements, such as doors, roofs, facades, beams, and windows, are annotated and used for training machine learning models on object detection. The heat loss U-values for building envelopes and elements are quantified in the evaluation layer, while influential parameters, such as the emissivity and reflected temperature, are analyzed.

### 3.2. Dataset Preparation

The number of objects in the dataset are not distributed equally due to the nature and context of the dataset itself. The frequency of HVACs and doors are far lower when compared to windows and facades for any given building, causing lower detection limit discrepancies for these respective objects. Two tactics were employed to remedy this issue: (1) Each dataset had several augmentation techniques applied to them. These included random color shifts, multiplying the dataset with copies of itself, horizontal and vertical flips, Gaussian blur, and contrast and brightness. The augmentation resulted in the original dataset increasing by a factor of six, on average. (2) Once augmented, datasets were combined based on campus buildings.

This technique drastically increased the objects with low occurrences, and allowed the model to learn and identify these objects more accurately. Each training dataset is listed in Table 3, and a total of four datasets were created for training. Datasets 1 through 3 consisted of images taken at the Museum of Art and Twamley buildings on the UND campus. Dataset 4 consisted of a combination of the Minot State, Wahpeton State, and Bismarck State campuses. The number of augmented instances of facades, windows, roofs, HVACs, and doors are listed in each column of Table 3. The total number of images used was 42,439.

### 3.3. Thermal Hotspot Detection via Clustering Techniques

#### 3.3.1. Threshold-Based Clustering (TBC)

**Figure 3:**
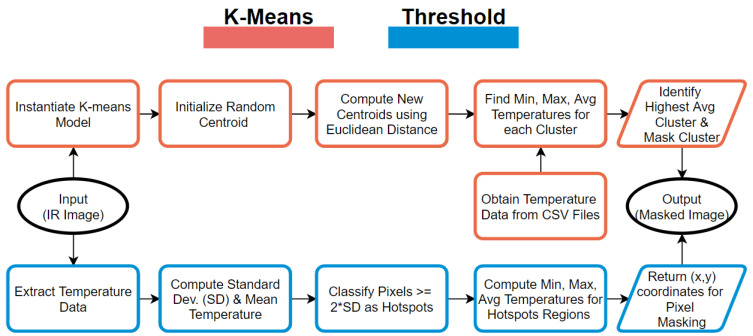
Flowchart of thermal image clustering using K-means and Threshold-Based Clustering.

Once the window- or facade-only pixels were obtained for a particular image, they were classified as areas of interest and passed through a threshold to obtain hotspot relevance. The initial testing of this approach applied the use of a static threshold based on percentiles; however, this approach was discontinued due to its inability to adapt and identify hotspots under extreme variations in the input surface temperatures. Figure 3 illustrates a flowchart for the two clustering algorithms.

Threshold-Based Clustering is based on the objects’ mean temperature (μ) and standard deviation (σ), considering object temperatures of >=2σ and processing the pixels corresponding to those temperatures. The following piece-wise functions can be represented, mathematically, as

(1) $$I_{x,y}=\{\begin{array}{cc} 1, & ift_{x,y}>=2\sigma +\mu \left[T_{o}\right]\\ 0, & ift_{x,y}<2\sigma +\mu \left[T_{o}\right] \end{array}$$

(2) $${\left(\hat{R},\hat{G},\hat{B}\right)}_{x,y}^{pixel}=\{\begin{array}{cc} {\left(255,0,0\right)}_{x,y}^{pixel}, & ifI_{x,y}=1\\ {\left(R,G,B\right)}_{x,y}^{pixel}, & ifI_{x,y}=0 \end{array}$$

where $I_{x,y}$ is the Identity Matrix that holds hotspot (binary 1) and non-hotspot (binary 0) pixels, $t_{x,y}$ is the pixel at co-ordinates (x,y), and $T_{o}$ is the set of all pixels within an object of interest, such as the walls or windows. ${\left(\hat{R},\hat{G},\hat{B}\right)}_{x,y}^{pixel}$ represents the RGB pixels at co-ordinates (x,y) that are colored red for a detected hotspot and unchanged if not.

The thresholds on surface temperature were evaluated using Infrared Camera Inc. (ICI) thermal imaging software. These thresholds are visually intuitive: when looking at the raw thermal image, the regions of longer wavelengths in the visible light spectrum, represented in red, are the areas with a greater density of pixels denoting a higher temperature. The user can identify these regions as hotspots and use a shaping tool to draw boundaries for segregating the image into hotspot sections. This activity is a tedious and inaccurate process that yields only the maximum, minimum, and average temperatures. These regions are identified on a granular level using TBC, where each pixel is analyzed for its suitability as a hotspot. Hotspot regions are higher temperatures, and due to heat dissipation, are considered regions of significance when estimating U-values.

#### 3.3.2. Hotspot Detection Using K-Means

K-means is a common data mining approach to group ‘N’ observations into ‘K’ clusters with the nearest mean, or centroid of a cluster, by minimizing the squared Euclidean distances. We evaluated groupings of surface temperature observations from the captured thermal images, which were each divided into ‘K’ clusters based on different colors formed by the combinations of color channels. The surface temperature observations were compared with TBC for the reliability of the clustering method. This method was created using the Scikit-learn Library [46].

K-means segments an image into different clusters based on colors. This approach is based on the idea that the colors in a thermal image represent different temperatures regions. This method can be further divided into two parts: segmentation and hotspot identification.

We calculated the minimum, maximum, and average temperatures for each cluster using the pixel temperature data from the CSVs when using hotspot identification. The methodology of the clustering phases is further explained in Algorithm 1.
**Algorithm 1:** Pseudo code for K-means Clustering.
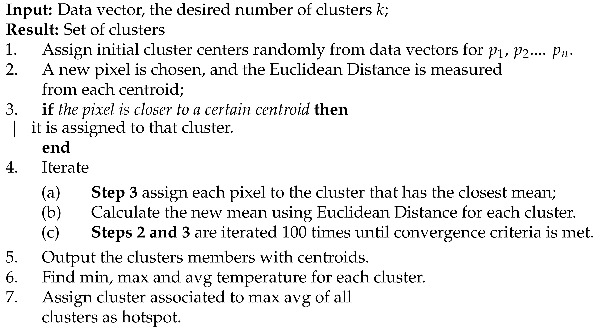


An image is a 3D vector of colors comprised of combinations of (i.e., Red, Green, and Blue) with value ranges from 0⟶255 for each channel. A cluster is determined by grouping those pixels with the least Euclidean Distance from the chosen centroid. Considering two pixels *i* and *j* with values ($R_{i}$, $G_{i}$, $B_{i}$) and ($R_{j}$, $G_{j}$, $B_{j}$). The Euclidean distance $D_{ij}$ between them can be further computed as follows:

(3) $$D_{ij}=\sqrt{{\left(R_{i}-R_{j}\right)}^{2}+{\left(G_{i}-G_{j}\right)}^{2}+{\left(B_{i}-B_{j}\right)}^{2}}$$

K-means proceeds by selecting random pixels as centroids. This method of initializing the centroid has been reformed over the years with a better seeding method called K-means${}^{++}$ as stated by Arthur et al [47]. The main goal for K-means is to reduce the Sum of Squared Distance for each cluster at every iteration. This is achieved by selecting new pixels as centroids and calculating the Euclidean distance with their respective cluster members. Equation (4) shows that, for each cluster from n=1 to *N*, a cluster is chosen, and the Euclidean Distance between a pixel in *n* and the centroid is calculated. This is iterated over and over for every cluster *n* to minimize *J* by selecting new pixels $P_{n}$ and centroids $C_{n}$.

(4) $$J_{min}=\sum_{n=1}^{N}\sum_{P\epsilon n}^{}D_{C_{n}P_{n}}$$

Computing the K-means for such large datasets raises the question of selecting the range of clusters. This problem is solved using the Elbow Method [48], which considers the sum of distances between cluster centers (and their respective cluster members) versus the number of clusters. For each dataset, this had to be computed to obtain a range of *k* or an optimal number of clusters that are ideal for that data set.

### 3.4. U-Value Estimation

Various U-value measurement-based methods have been used in the literature to estimate thermal performance. According to the Stefan–Boltzmann law, the radiative heat varies with the fourth power of temperature difference [49].

The U-value estimation for the external surface was carried out while considering the wind velocity using the following equation in [50,51,52]

(5) $$\begin{array}{cc} \; & U_{1}=\frac{\epsilon \sigma \left(T_{se}^{4}-T_{ae}^{4}\right)+3.805v\left(T_{se}-T_{ae}\right)}{T_{ai}-T_{ae}} \end{array}$$

where *v* is the velocity of the external wind, $T_{se}$ denotes the external surface temperature, $T_{ai}$ denotes the internal air temperature, and $T_{ae}$ denotes the external air temperature. The radiative heat component is similar to the Stefan–Boltzmann equation [49] and the convective term is derived from Jargon’s formula [53].

**Figure 4:**
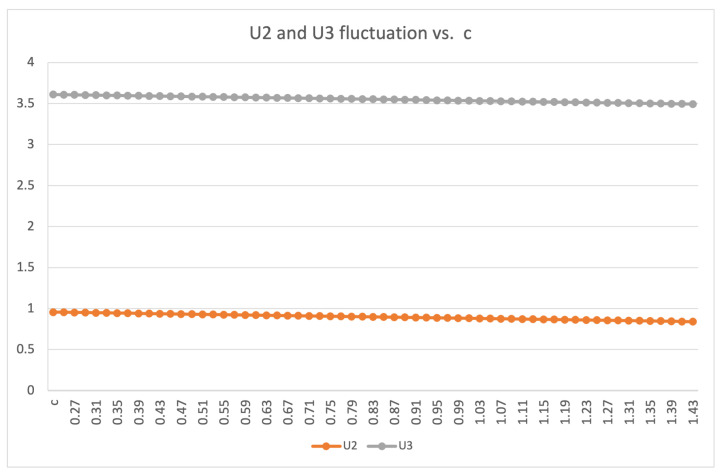
Calculated U-values based on variation of *c*${}_{1}$.

Fokaides et al. [54] estimated the U-value according to the Stefan–Boltzmann law as follows.

(6) $$\begin{array}{cc} \; & U_{2}=\frac{4\epsilon \sigma T_{s}^{3}\left(T_{si}-T_{refl}\right)+\alpha_{c}\left(T_{si}-T_{ai}\right)}{T_{ai}-T_{ae}} \end{array}$$

where the wall emissivity and convective heat coefficient is denoted by ε and $\alpha_{c}$, respectively. In addition, σ denotes the Stefan–Boltzmann constant and $T_{in}$, $T_{out}$, $T_{si}$, and $T_{refl}$ denote the internal, external, internal surface, and reflexive wall temperatures respectively. The surface temperature is denoted by $T_{s}$. The mean temperature was introduced in Equation (7) by Madding et al. [55] to replace the surface temperature in Equation (6) to estimate the U-values as follows:

(7) $$\begin{array}{cc} \; & U_{3}=\frac{4\epsilon \sigma T_{m}^{3}\left(T_{si}-T_{refl}\right)+\alpha_{c}\left(T_{si}-T_{ai}\right)}{T_{ai}-T_{ae}} \end{array}$$

where $T_{m}=\frac{T_{s}+T_{refl}}{2}$ denotes the mean temperature, and ϵ and σ denote the emissivity and Stefan–Boltzmann constant, respectively. In this equation, the reflective temperature is subtracted from the surface temperature. Spectrum emissivities of 0.75 for the wall and 1.0 for windows were taken. The material for walls was considered to be fire brick, and the windows were classified to be of a smooth glass material, which had emissivities in the range of 0.92–0.96 [55,56,57]; however, for the purposes of this paper, glass was considered to be a perfect black body (in the context of U-value estimation) as there would be negligible differences if we use Equation (5) to calculate the U-values. The convective coefficient $\alpha_{c}$ depends on various factors, including the height of the wall and the temperature difference shown in Equation (8).

(8) $$\begin{array}{cc} \; & \alpha_{c}=c_{1}{\frac{\left(T_{si}-T_{ai}\right)}{L}}^{\frac{1}{4}} \end{array}$$

where *L* is the height of the wall and the value of $c_{1}$ varies from 1.31 to 1.42. Equation (8) is obtained from reference [55], where $c_{1}$ varied from 0.25 to 1.42 depending on the type of air-flow. When the wall is tall, and the temperature difference is large, the coefficient can change depending on the type of flow (turbulent or laminar). Figure 4 illustrates the deviation of the U-value with respect to acceptable *c*${}_{1}$ values within the range of 0.25 to 1.42. The acceptable range of *c*${}_{1}$ values was deduced empirically. Several types of objects and their corresponding U-values were plotted with *c*${}_{1}$ values varying from 0.25 to 1.42. These U-values were then converted to BTU/hr ft${}^{2}$°F first and then to W/m${}^{2}$·K before being compared to ASHRAE standards, which allowed us to identify which constants aligned with the expected values.

We consider $U_{c}$ as the average of the U-values, calculated previously as

(9) $$U_{c}=\frac{U_{1}+U_{2}+U_{3}}{3}$$

The IR Flash Pro software was used to extract the temperature information into a CSV file containing 512 × 640 cells. The building height information was obtained from the architectural diagram.

## 4. Results and Discussion

In this section, examples of results are presented and discussed. We start by presenting an evaluation of computer vision algorithms for detection and instance segmentation. Then, we present some examples of results related to clustering and their analysis as well as their discussion. In the last part, we present the U-value estimation as well as examples of the obtained results using different formulas.

### 4.1. Evaluation Metrics

In order to evaluate the performance of deep-learning-based thermal image instance segmentation, a confusion matrix can be used, and, from this, several other metrics can be derived. Table 4 shows the confusion matrix and is defined to show the model’s ability to correctly and incorrectly identify objects.

One of the popular metrics used for measuring the accuracy of object detection is the average precision (AP). The average precision computes the AP value for a recall value of 0 to 1. The precision quantifies the percentage of correct predictions. Recall measures how well the positive values are detected. The mathematical definitions of precision and recall are as follows:

(10) $$Precision=\frac{TP}{\left(TP+FP\right)}$$

(11) $$Recall=\frac{TP}{\left(TP+FN\right)}$$

where *TP* is true positive, *FP* is false positive, and *FN* is false negative.

**Figure 5:**
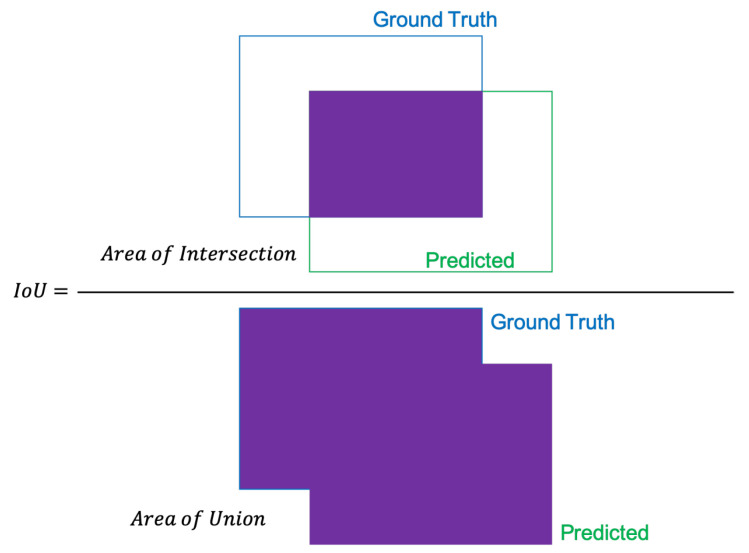
Computing the Intersection of Union, *IoU*, calculated by dividing the area of overlap between the bounding boxes by the area of union.

In order to determine true positives, the intersection over union is used (Figure 5). The *IoU* measures the area of overlap between the ground truth and prediction boundaries. Mathematically, the intersection over union is calculated as the ratio of the area of the overlap to the area of union.

(12) $$IoU=\frac{A_{o}}{A_{u}}$$

where $A_{o}$ and $A_{u}$ are the areas of overlap and union respectively. If the IoU is greater than the threshold, the detection is considered correct, otherwise, it is a false detection.

The general definition of the average precision is finding the area under the precision–recall curve.

(13) $$\int_{0}^{1}p\left(t\right)dt$$

The interpolated AP is calculated by replacing p(t) in Equation (13) by

(14) $$\int_{0}^{1}\max_{r\geq t}\left(r\right)dt$$

### 4.2. Results of Detection and Instance Segmentation Based on Deep Learning

The models were trained on a machine containing an Intel Core i9-9920X with four Nvidia GeForce RTX 2080 Ti GPU’s. Each card consists of 11 GB GDDR6 memory and 544 tensor cores. Each model was trained on one GPU with different configurations, and the model with the best metrics was chosen to be trained on by the next dataset. Adjusted configurations include the batch size, learning rate, and epochs. Table 5 illustrates the learning rate, number of epochs, and training time for each dataset. For the first three datasets, we noticed that reducing the learning rate by a factor of ten at each subsequent training session helped to improve the model accuracy.

This improved model accuracy was due to the first three datasets containing data from UND campus buildings, which have similar architectures. Dataset four consisted of several different campuses, and thus a higher learning rate yielded better results. The training time for each dataset was proportional to the number of images found within them. Dataset four consisted of three different campuses, since training on each individual campus degraded the model performance.

The test dataset was generated with 10 images from each campus. These 70 images were subsequently deleted from their original datasets to eliminate them from the machine learning process. Similar augmentation techniques were applied to the test dataset to increase the size and test the models fitness. After the augmentation process, the size of the dataset increased to 213 images. The breakdown of dataset is provided in Table 3, which breaks down each dataset by the number of instances in each class within it along with the cumulative values.

Table 6 shows the average precision and the mIoU of the three object detection models and one instance segmentation model. The models were trained on and validated using thermal images captured by the ICI Mirage 640 camera and the ratio to training verses testing was 90:10. A total of five classes were identified for the models to train on: Windows, Facades, Roofs, HVACs, and Doors. The models were evaluated after each training session; however, the results presented are after the final training session. The Average Precision at thresholds of 25%, 50%, and 75% were recorded, and the results show that Mask R-CNN outperformed the other three models for all thresholds. The other three models especially suffered at the 75% threshold, which indicates that the models are only able to identify a few objects with high confidence.

The feature maps generated were not adequately able to capture the patterns in this thermal dataset leading to low confidence in the models. The three object detection models suffered in estimating the size of objects as well. This is shown in the low mIoU scores achieved by the models. It is also beneficial to compare the pure object detection models against themselves. All three object detection models utilized Faster R-CNN with different backbone architectures.

These models were also evaluated to a similar AP score at all thresholds; however, the Inception ResNetV2 backbone performed slightly better. This is prevalent in the slightly higher AP at 0.75. The mIoU of both the Inception ResNetV2 and ResNet 50 were the same at 0.34; however, the Inception ResNetV2 backbone achieved higher results for windows, roofs, doors, and HVAC systems while the ResNet 50 model achieved a higher facade evaluation. Overall, Mask R-CNN achieved an average mIoU of 0.66 with Facade and Roofs having the highest overlap of 0.73 and 0.67, respectively.

The Mask R-CNN model was selected for two main reasons. When quantifying heat loss on buildings, the U-value equations are extremely sensitive to small shifts in temperature and emissivity. This sensitivity required our detection to be precise, with traditional bounding box detection being insufficient for our purposes. Using bounding boxes allows for noise to be introduced since the object contour is not calculated. Instance Segmentation allows us to classify accurate results in greater detail to match ASHRAE standards. Emissivity plays a large role within each of the U-value equations and changes based on the material composition of the object in question. Based on the classification and composition, the emissivity value was looked up on multiple infrared emissivity tables.

**Figure 6:**
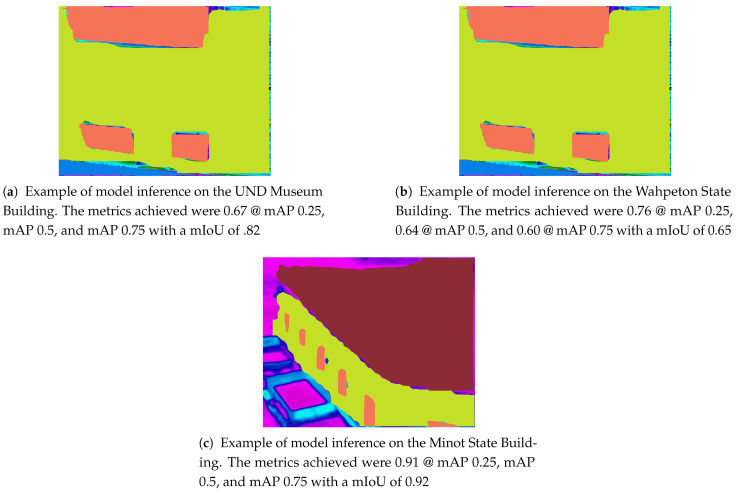
Examples of images of building segmented using Mask-RCNN trained on the heat loss dataset.

The Mask R-CNN model also yielded better results (please see Figure 6) when compared to the Faster R-CNN models with different backbones. Both object detection and instance segmentation models were trained in a similar fashion with varying learning rate decay for the first three datasets, and higher decay for the fourth dataset. The number of epochs was held constant for all models. With the introduction of the mask branch, the Mask R-CNN model took longer to train with more favorable results. We, therefore, selected the Mask R-CNN model.

### 4.3. Clustering Performance

Metrics, such as the Silhouette Coefficient and Davis–Bouldin Index, were evaluated for K-means. As explained by [58], the Silhouette Coefficient is a popular metric to find the quality of clustering. It is a measure of how a particular data point or pixel value in our use case is similar to its own cluster compared to other clusters. The coefficient ranges from −1 to 1, where a positive value signifies that the clustering was well performed. Davis et al. [59] introduced the Davis–Bouldin Index. This metric is an average of the similarity for a cluster to its nearest cluster, which is a ratio of the intra-cluster distance to the inter-cluster distance. The minimum score is 0, with lower values indicating better clustering. The Silhouette Coefficient for the Museum of Art and Twamley buildings were 0.71 and 0.68, respectively. The Davis–Bouldin Index for the Museum of Art and Twamley buildings dataset were 0.81 and 0.75, respectively.

**Figure 7:**
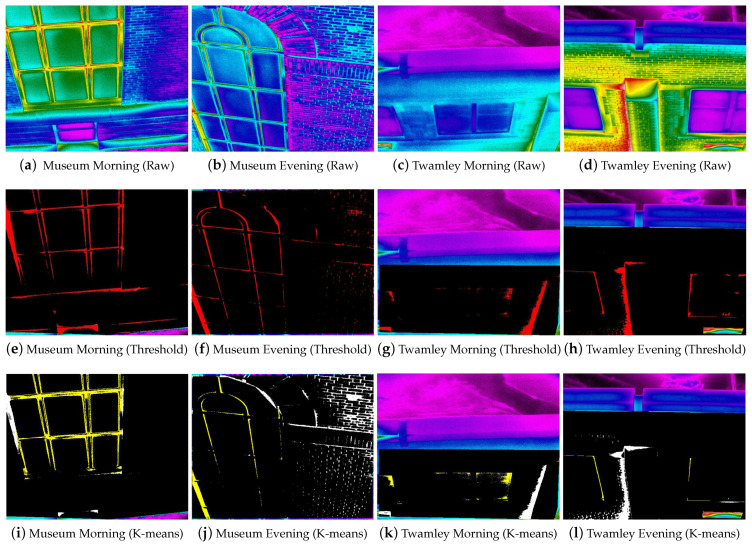
Raw (**a**–**d**) and hotspot (**e**–**l**) images for two particular windows of Museum and Twamley over the morning, afternoon, and evening.

Figure 7 highlights the hotspot regions in discrete red and yellow sub-regions for a window (Window 1) at the UND Museum of Art and Twamley buildings using the TBC and K-means approaches, respectively.

Table 7 compares the two clustering methods and establishes a comparison metric (called the overlap) for windows and facades, respectively. The overlap metric is the ratio of overlapped hotspot pixels or similar pixels identified individually by the Threshold and K-means approaches to the total number of hotspot pixels identified by each of the clustering approaches. Keeping a maximum error of 10%, there were five instances when the two clustering methods can be considered to be in agreement. However, this is marginally short of a 50% split and cannot be used to definitively conclude a consensus. Two other metrics were considered for comparison and are discussed in the following two paragraphs.

**Figure 8:**
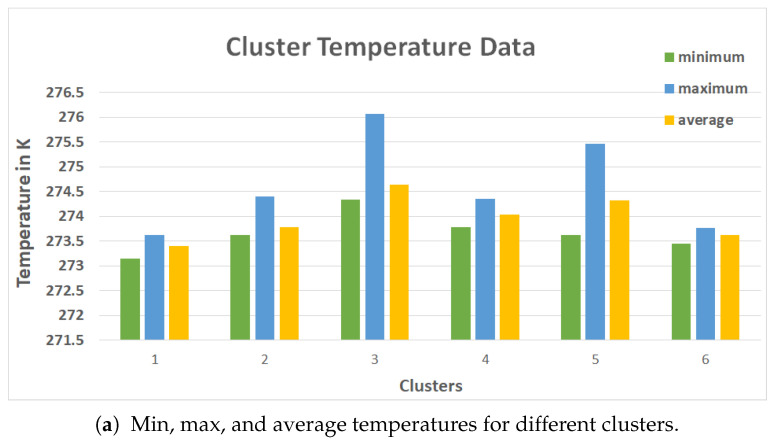

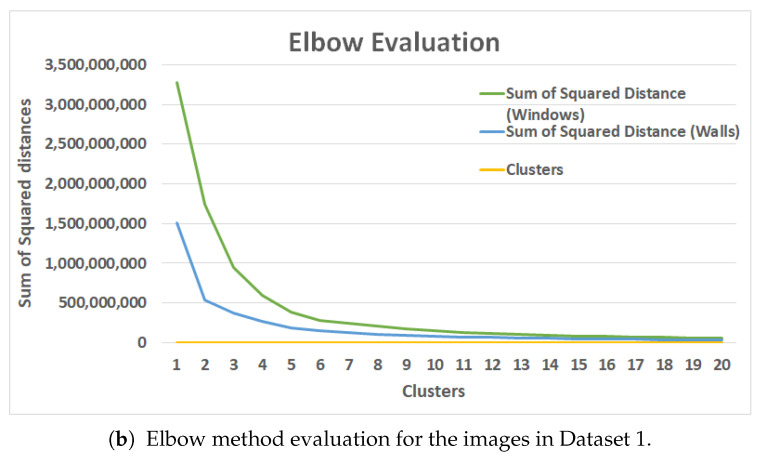
The K-means-optimal cluster evaluation and temperature results.

Figure 8b depicts the minimum, maximum, and average surface temperatures for six clusters created in the segmentation phase. The cluster with the highest average temperature (such as cluster 3 Figure 8b) was chosen as the hotspot. Figure 8b shows the Elbow evaluation for an image from the museum dataset. From the graph in Figure 8, we can identify the *k* value for the walls to be somewhere in the range between 3 to 6. After the seventh cluster, it was evident that there were no such changes in the squared distance. The K value of six was chosen using the temperature TBC as the ground truth because of the hotspot evaluation technique involving pixel temperatures. Computing the K-means to six clusters yielded results with few deviations with respect to Average Hotspot Temperature and Density of Hotspot from TBC. A value of K = 5 yielded results similar to the TBC for the windows. The performances of the clustering techniques across different parameters are listed in Table 8.

Table 8 compares the results obtained by the clustering approaches based on a fixed set of five parameters across the morning, afternoon, and evening time periods. The “Density (Hotspot)” measure is a ratio of the number of hotspot pixels to the total number of pixels within the entire surface being measured, such as windows or facades. Similarly, the “Average Temperature (Hotspot)” measure is the average temperature of the hotspot regions identified by each of the clustering methods. The largest discrepancy can be seen when comparing the density metric between the two clustering approaches during the afternoon for Twamley. This discrepancy was caused by the incidence of solar radiation on Twamley’s surface.

For a fair comparison, the average hotspot temperatures across different time frames can be taken into account. The values obtained for these measures are consistent across the morning (both the buildings), afternoon (museum only), and evening (both the buildings) time periods with average hotspot temperature differences between 0.01 and 0.48 degrees Kelvin and can be considered negligible. The afternoon duration for Twamley is not considered because, as mentioned earlier, a skewing factor was introduced by solar irradiance. It should also be noted that the temperature values obtained by the sixth cluster from the K-means approach were the most accurate values for the Museum dataset. Accuracy here was assessed when the values of the K-means approach were closest to the values from the TBC as temperature values in the latter were extracted directly from each pixel and, thus, are taken to be the ground truth.

In order to obtain the U-value for UND’s buildings, the Stephen–Boltzmann constant σ was replaced by 5.67 × 10${}^{-8}$ Wm${}^{-2}$ K${}^{-4}$ in Equation (5) in addition to the spectrum emissivities mentioned earlier. Table 9 and Table 10 show the U-values and related parameters for UND’s Museum and Twamley buildings). Each of these tables contains the investigated building elements, number of images considered, min–max–average surface temperature captured from the thermal images, air temperature obtained from weather data, $U_{1}$, $U_{2}$, $U_{3}$, and $U_{c}$ (first obtained in BTU/ft${}^{2}$ h°F and then converted to W/m${}^{2}\cdot$K and where $U_{c}=\frac{U_{1}+U_{2}+U_{3}}{3}$) using the corresponding equations and ASHRAE standard data.

The thermocouple temperatures obtained from the building surface were through an Extech 3-channel data logger, which had conductive probes to measure surface temperatures. These probes were secured to the indoor and outdoor surfaces using electrical tape for average durations of 20–30 s to obtain a steady reading of the surface measured. Different points on the surface were used, and, if the temperature readings did not differ too greatly from one another within that time frame, average values were taken.

According to the results obtained from the thermal images, the single-pane window U-values (Twamley building) were always more than the double-pane window (Museum building) U-values due to the fact that the double-pane windows consist of an extra layer of air that acts as an insulation to the heat flow. It can also be observed that the wall 1’s (in Table 9) *U*${}_{1}$ values are more consistent with the ASHRAE standard while window 1’s *U*${}_{2}$ and *U*${}_{3}$ values in Table 10 (highlighted in green) are more consistent with the ASHRAE standard than *U*${}_{1}$. As there are many factors that influence U-value estimation (please see the following subsection on uncertainty analysis), additional testing needs to be done through rigorous data collection (multiple time frames, precise indoor temperature readings, varied building types, etc.) to come to accurate conclusions.

### 4.4. Factors Contributing to Uncertainties in Thermal Data Capture and Processing

The proposed approach consists of three primary layers: (1) the collection of data and instance segmentation using deep learning; (2) clustering and hotspot detection; and (3) U-value estimation. These three layers contribute to the overall uncertainties of the proposed solution. In the following, we discuss each of these points:

1. Uncertainties associated with image capturing include the following:
  - Capturing images of surfaces during the daytime should be planned carefully since solar irradiance can skew readings from the imaging apparatus [60]. Sunlight reflecting on external surfaces, such as brick, which is of high emissivity, will radiate more heat than if the surfaces were under shade. We used images obtained before sunrise and after sunset; however, the effects of incident sunlight will still affect the surface for hours after the surface is shaded.
  - Surrounding objects, such as metallic surfaces, may reflect high temperatures, leading to inaccurate surface measurements due to reflecting sunlight [61]. We minimized this bias, recognizing that the buildings in these datasets are adjacent to parking lots, which had vehicles with reflective surfaces. These reflections will influence the thermal readings.
  - Heat and humidity are two atmospheric factors that will influence temperature readings [62]. In regions where the temperatures and relative humidity fluctuate quite frequently, measurements must be systematically recorded when there is acceptable consistency in weather patterns for that day or time.
2. Uncertainties with object detection and instance segmentation: Uncertainty in deep learning can be classified mainly into two types: epistemic uncertainty and aleatory uncertainty. Epistemic uncertainty refers to the uncertainty associated with the objects that the model does not know because the training data was not appropriate. This type of uncertainty arises due to gaps in data and knowledge. We limited this type of uncertainty by generating sufficient data as this results in decreasing epistemic uncertainty. The aleatory uncertainty refers to the type of uncertainty rising from the stochasticity of the observations. This second type of uncertainty cannot be mitigated by providing more data to the models. Given the uncertainty in deep learning, the reading of the data associated with U-value calculation is subsequently uncertain, and there will be some variability the readings and the overall U-value estimation. These variabilities are added to other factors discussed in the previous paragraph.
3. Uncertainties with clustering and hotspot detection: The clustering and hotspot detection are directly related to object detection and instance segmentation and uncertainty associated with deep learning will propagate and create uncertainties associated with this part. Apart from these sources of uncertainty, additional sources exist, such as the observations, background knowledge, the induction principle, and the learning algorithm used for this unduction principle.
4. Uncertainties with U-value estimation: The formulas used for U-values are approximations and depend on many factors that are themselves subject to different types of uncertainties, which can result in different measurements.

Please see Table 11 for quantitative reporting of the precision and average deviation when considering U-value estimation. Based on the results from our analysis and due to the high number of sample points for object-wise U-value estimation, unbiased rounding was used to retain one significant digit after the decimal for precision and error, and two significant digits after the decimal for the average deviation. For instance, the wall precision value for Twamley and the error in the wall readings for the Museum were rounded to 15.1% and 347.9% from 15.08% and 347.91%, respectively. For the purposes of our evaluation, we specify the definition of precision according to ISO 3534-1 [63] to be “the closeness of agreement between independent test results obtained under stipulated conditions.”

The error was calculated by considering the % difference between the empirical observations and the true values (ASHRAE) [64]. This can be considered to be a measure of accuracy. Following the standard definition for “true value”, the “true value” refers to values obtained by ASHRAE (which may have had systematic or random uncertainties) and not the absolute value for the measurand that is devoid of any contributing or biasing factors. The average deviation ($\Delta U_{avg}$) is calculated using the following formula:

(15) $$\Delta U_{avg}=\sum_{i=1}^{3}\frac{|U_{i}-U_{c}|}{3}$$

where *U*${}_{i}$ represents the U-values 1, 2, and 3. The precision is calculated using Equation (16)

(16) $$Precision=\frac{\Delta U_{avg}}{U_{c}}\times 100\%$$

As can be seen from Table 11, the average deviation for windows was equal to or higher than those of walls for both the buildings. This means that the variation of U-values from their respective average value (*U*${}_{c}$) for a given object was lower in the case of walls than windows. We can infer from this table that the U-value measurements for walls were much more similar to one another relative to the windows’ U-values.

Similarly, in terms of accuracy, the U-values obtained for the windows are closer to the true ASHRAE values. These results also confirm an important result: U-values closer to one another may not necessarily indicate higher accuracies as can be seen when the accuracy for walls are considered. Using our methodology for U-value estimation and when considered relative to windows, it can be said that the measured values for walls are more precise (lower precision) but much less accurate (higher errors).

## 5. Conclusions

Building thermal performance information is crucial to reducing energy consumption and to achieving zero energy buildings. Researchers have proposed many methodologies over the past decades, including statistical approaches, engineering-based methods, and machine learning. These methods present many limitations; therefore, this study aimed to enhance the building thermal performance with a more precise heat loss quantification and to overcome the complexity of engineering methods.

We proposed a novel method using thermal imagery and deep-learning-based instance segmentation combined with analytical methods to compute U-values. We used thermal images captured by SkySkopes to train the machine learning models. The images were obtained during several flight rounds duringearly dawn to avoid any non-desirable reflections and accounted for several variables, such as the angle and distance to walls. The images obtained were annotated and archived using cloud storage. Several classes were defined, such as the facades of buildings, trees, and windows, after which Mask R-CNN was trained and tested.

The confusion matrix and AP were computed to evaluate the performance of the machine learning algorithms. The results indicated that the model trained on augmented datasets achieved total average precision values as high as 79% for facades, 69% for windows, and 67% for roofs. The heat loss calculation was also used to quantify the desired values. We proposed clustering and hotspot detection methods to identify the primary regions of heat loss in the facades and windows of the buildings.

Three measures were used to compare the clustering schemes. The overlap metric indicated a 50% agreement between the methods; however, we explored the average hotspot temperature metric to obtain a definitive conclusion. A maximum difference of 0.48 degrees was observed for the average hotspot temperature metric on surfaces not affected by sunlight and, thus, was effectively used to confirm our results. This information can be leveraged to make appropriate decisions related to building design and maintenance.

The analysis led to the following conclusions: (1) the proposed data driven approach provided an automatic and reliable process for energy audit applications; (2) our results are broadly consistent with the American Society of Heating, Refrigerating, and Air-conditioning Engineers building standards; (3) this research generated new information on the dependency of thermal efficiency, which relies on many factors, including the thermal images acquisition process, building geometry, and indoor heating or cooling conditions; and (4) the findings of this research and the quantitative and qualitative uncertainty analyses will provide a significant starting point for discussion and further research in the area of automated processes for energy audit applications.

Future work will include re-working Mask R-CNN to analyze more than thermal images and with datasets consisting of more balanced classes. Further studies should investigate the possible effects of the building typologies on the meteorological performances of the proposed method.

## Figures and Tables

**Table 1:** State-of-the-art computer vision techniques.

Computer Vision Task	Model	Structure/Backbone	Metrics	Comments
Detection	R-CNN [18,19]	Selective Search Algorithm + SVM	mAP of 62% on PASCAL VOC 2012	Slow because of the high number of region proposals (2000) (47 s/test image)
	Fast R-CNN [20]	Selective Search Algorithm + FCs	mAP of 39.3%@0.5 on MS COCO and 66% on PASCAL VOC 2012	“Fast compared with R-CNN 0.32 s/testing image Fast R-CNN trains the very deep VGG16 network 9 × faster than R-CNN, is 213 × faster at test-time”
	Faster R-CNN [21,22,23]	Region Proposal Network + ROI pooling + FCs	mAP of 42.7% on MS COCO and mAP of 78.8% PASCAL VOC 2012	Remove Selective Search Algorithm
	SSD	ResNet101 + FCs	mAP of 31.2%	Runs at 125 ms
Real-Time Object Detection	YOLO [24,25]	Single Regression from image pixel to class BB (Darknet-53)	YOLOv3-320 mAP of 28.2%, YOLOv3-416 mAP of 31%, and YOLOv3-608 mAP of 33%”	Support real-time (up to 45 FPS for YOLOv3-320)
	DeconvNet [26]	ConvNet (VGG-16) concatenated with DeconvNet	mAP of 70% on PASCAL VOC 2012	
	FCN [27]	FCN introduces the skip connection to fuse feature layers of different scales	Graph-FCN achieves mIoU of 65.91% on PASCAL VOC Dataset and FCN-32 achieves mIoU of 36.64% on PASCAL-Context	None
Semantic Segmentation	ParsNet [28]	Improved FCN	ParseNet Baseline achieves mIoU of 67.3% on PASCAL VOC Dataset and ParseNet achieves mIoU of 69.8% on PASCAL VOC Dataset	
	Deeplab [29]	Atrous Convolution for Dense Feature Extraction + Atrous Spatial Pyramid Pooling + Fully-Connected Conditional Random Fields	DeepLab achives mIoU of 64.96% on PASCAL VOC 2012 and DeepLab-LargeFOV achieves mIoU of 65.82% on PASCAL VOC 2012	Objective function is optimized in all layers with respect to weights by SGD standards. Imagenet classifier is replaced with classifier equaling number of target classes in last layer.
Instance Segmentation	EncNet [30]	ResNet + Context Encoding Module	mIoU of 52.6% on PASCAL-Context Dataset	Introduces very little extra computation to original FCN network. Context Encoder is light weight.
	Mask R-CNN [20,31,32]	RPN + ROIAlign + Mask + ResNet101	mAP of 39.8% and 63.1% on MS COCO for keypoints & mask	Replaces ROI pooling with ROIAlign in Faster R-CNN architecture and includes FCN for segmentation
Panoptic Segmentation	Machine panoptic segmentation [33]	Unified semantic segmentation and instance segmentation	$PQ^{th}$ of 54%, 79.4%, 67.8% on Cityscape	Unique evaluation methodology

**Table 2:** State-of-the-art image clustering techniques.

Clustering Method	Performance	Reference
K-means/DBSCAN	K = 15 was optimal for accuracy and computation time. Further segmentation by DBSCAN yielded 136 clusters for precision.	[37]
K-means/Threshold	K = 4 was optimal and yielded a 99.7% accuracy rate in hotspot detection for an ensemble ML model called Voting (Naive Bayes + REPTree).	[38]
Dual Clustering Scheme	Precision and recall produced averages of 80% and 58.3% respectively for 10 sets of images.	[39]
IFS/Fuzzy C-means	Average segmentation and Dice scores of 99% for varying levels of noise corrupted images.	[40]
IP-MS	Average of 1.4 s per sample image in contrast to 2.3 s from the K-means algorithm. Higher accuracy than K-means in terms of blue color intensity representations.	[41]
DEMP-k (Directly Estimated Misclassification Probabilities)	Tested on digit recognition gives correct classification (CP) = 0.843, adjusted Rand Index (AR) = 708 and computing time (τ) = 27.03 s	[42]

**Table 3:** Dataset breakdowns.

Datasets	# of Facades	# of Windows	# of Roofs	# of HVACs	# of Doors	Total Images
Dataset 1	2060	1109	634	343	100	2562
Dataset 2	10,190	13,987	1894	0	126	10,971
Dataset 3	2576	5207	492	2085	282	2541
Dataset 4	26,217	18,684	11,747	1616	6448	26152
Test Dataset	207	176	95	38	43	213
Total	41,250	39,163	14,862	4082	6999	42,439

**Table 4:** Confusion matrix.

	Positive (1)	Negative (0)
Positive (1)	TP	TN
Negative (0)	FP	FN

**Table 5:** Hyper parameters of the best performing model at each training session (Mask R-CNN).

Training Dataset	Learning Rate	Epochs	Training Time
DS1	0.001	75	7 h 35 m
DS2	0.0001	100	30 h 38 m
DS3	0.00001	150	21 h 47 m
DS4	0.0001	200	288 h 35 m

**Table 6:** The average precision of computer vision algorithms trained and tested on OGI thermal images with three different threshold values for five classes: windows, facades, roofs, HVACs, and doors.

Classes	${AP}^{0.25}$	${AP}^{0.5}$	${AP}^{0.75}$	Intersection	Union	mIoU
Window	0.39	0.39	0.18	12,648.28	27,679.36	0.45
Facades	0.34	0.31	0.12	39,487.61	118,876.24	0.33
Roof	0.41	0.31	0.07	20,042.73	55,555.64	0.36
HVAC	0.27	0.27	0.09	192.43	1085.39	0.17
Door	0.06	0.06	0	414.12	6955.88	0.05
Faster R-CNN Inception ResNetV2	0.29	0.27	0.09	15,503,247	44,762,491	0.34
Window	0.26	0.10	0.03	10,011.70	28,303.41	0.35
Facades	0.41	0.32	0.05	29,035.47	126,352.91	0.22
Roof	0.38	0.26	0.13	16,002.26	59,677.54	0.26
HVAC	0.09	0	0	314.15	1927.03	0.16
Door	0.35	0.26	0	674.93	6983.51	0.09
Faster R-CNN Inception V2	0.30	0.19	0.04	11,936,209	47,551,061	0.25
Window	0.28	0.19	0.12	10326.40	29,569.77	0.34
Facades	0.31	0.26	0.07	49,439.15	129,971.74	0.38
Roof	0.42	0.16	0.012	16,804.96	53,316.74	0.31
HVAC	0.23	0	0.012	117.57	1011.42	0.11
Door	0	0	0	411.18	8357.96	0.04
Faster R-CNN ResNet 50	0.25	0.20	0.07	16,422,148	47,334,488	0.34
Window	0.70	0.69	0.44	21,545.78	39,170.45	0.55
Facade	0.81	0.79	0.67	131,982.81	179,617.92	0.73
Roof	0.67	0.67	0.67	53,879.35	80,260.40	0.67
HVAC	0.27	0.27	0.18	508.23	4501.80	0.11
Door	0.67	0.67	0.68	2815.73	11,665.11	0.24
Mask R-CNN	0.62	0.62	0.53	14,961,967	22,380,315	0.66

**Table 7:** Hotspot pixel overlapping (%) for windows and walls.

Object of Interest	Windows				Walls			
**Buildings**	**Museum**		**Twamley**		**Museum**		**Twamley**	
**Duration**	**Threshold**	**K-Means**	**Threshold**	**K-Means**	**Threshold**	**K-Means**	**Threshold**	**K-Means**
**Morning**	88.2%	64.2%	86%	93%	69.8%	76.8%	79%	70%
**Evening**	82.8%	71.8%	43%	93.4%	71.4%	42.1%	72%	40.4%
**Afternoon**	77.9%	73.1%	82%	43%	72.8%	64.2%	34%	35%

**Table 8:** Performance of K-means and Threshold-Based Clustering.

Object of Interest		Walls				Windows				
Buildings		Museum		Twamley		Museum		Twamley		
**Morning**	**No. of Pixels (Object)**	197,186	197,186	186,601	186,601	36,167	366,167	17,926	17,926	280.35 K
	**Density (Hotspot)**	3.72	4.71	2.84	4.15	5.18	8.42	3.56	3.39	
	**No. of Pixels (Object)**	7241	9266	5340	8165	2022	3560	4383	4098	
	**Avg. Temp (Hotspot)**	269.71	270.08	270.09	270.15	268.91	268.8	268.72	268.71	
	**Avg. Temp (Object)**	268.48	268.48	268.7	268.7	268.04	268.04	268.17	268.17	
**Evening**	**No. of Pixels (Object)**	200325	200,325	183,642	183,642	42572	42,572	24,204	24204	273.25 K
	**Density (Hotspot)**	1.89	6.25	1.19	5.15	5.43	7.37	4.31	1.48	
	**No. of Pixels (Object)**	3340	12,526	3410	9973	2626	2802	999	417	
	**Avg. Temp (Hotspot)**	277.27	276.98	279.24	278.81	274.36	274.32	273.66	274.14	
	**Avg. Temp (Object)**	275.48	275.48	276.54	276.54	273.31	273.31	272.21	272.21	
**Afternoon**	**No. of Pixels (Object)**	202409	202,409	177,711	177,711	138,911	139,811	33,185	33,185	276.25 K
	**Density (Hotspot)**	2.99	5.66	1.19	5.76	4.7	6.22	2.18	7.31	
	**No. of Pixels (Object)**	6221	11,560	2026	9984	7023	9900	775	1384	
	**Avg. Temp (Hotspot)**	294.28	294.12	308.58	321.25	281.7	281.32	319.68	317.33	
	**Avg. Temp (Object)**	283.9	283.9	303.46	303.46	278.86	278.86	304.2	304.2	

**Table 9:** Museum U-value estimation (morning) on 17 March 2020.

Building Elements	# of Images	Temperature Analysis					U-Value Analysis (W/m${}^{2}$·K)				ASHRAE
		Surface Temperature (K)			Thermocouple	External Air					
		Max	Min	Avg	Temperature	Temperature	*U*_1_	*U*_2_	*U*_3_	*U*_c_	
**Window 1**	19	272.39	267.36	268.22	268.45 K	266.15 K	0.73	1.98	1.96	1.53	1.98
**Window (all)**	321	277.27	266.15	268.5			1.53	3.50	3.46	2.83	1.98
**Wall 1**	435	278.8	265.85	268.53			1.41	2.59	2.55	2.15	0.48
**Roof**	11	269.55	266.5	267.25			0.68	3.46	3.4	2.27	0.22

**Table 10:** Twamley U-value estimation (morning), 17 March 2020.

Building Elements	# of Images	Temperature Analysis					U-Value Analysis (W/m^2^·K)				ASHRAE
		Surface Temperature (K)			Thermocouple	Air					
		Max	Min	Avg	Temperature	Temperature	*U*_1_	*U*_2_	*U*_3_	*U*_c_	
**Window 1**	150	271	267	268.3	281.05 K	266.15 K	1.13	3.52	3.46	2.66	5.39
**Windows (all)**	500	279.25	266.83	268.25			1.36	3.46	3.46	2.78	5.39
**Wall 1**	45	281	266.08	269			1.79	2.61	2.55	2.32	0.48
**Roof**	11	270.3	266.7	267.4			0.73	3.46	3.46	2.55	0.22

**Table 11:** The error, precision, and deviation for U-value estimations.

Building	Object of Interest	Error (%)	Precision (%)	$\Delta U_{\mathrm{avg}}$	ASHRAE Standard
**Twamley**	Wall	383.3	15.0	±0.35	0.48
	Window	48.4	33.3	±0.93	5.39
**Museum**	Wall	347.9	24.5	±0.53	0.48
	Window	43.0	30.6	±0.87	1.98
	Window 1	22.7	36.6	±0.56	1.98

## Data Availability

Data can be made available on request due to privacy restrictions. The data presented in this study are available on request from the corresponding author. The data are not publicly available due to a binding legal contract with a private firm (registered name mentioned below in “Acknowledgments”) which was responsible for acquiring the data.

## Abbreviations

The following abbreviations are used in this manuscript:

CNN	Convolutional neural network
IoU	Intersection over union
ROI	Region of interest
YOLO	You Only Look Once
OGI	Optical gas imaging
FLIR	Forward looking infrared
HVAC	Heating, ventilation, and air conditioning
R−CNN	Regions based Convolutional neural network
MaskR−CNN	Region neural network
AP	Average precision
MAP	Mean average precision
TP	True positive
FP	False positive
TN	True negative
FN	False negative
$U_{1}$	U-value using formula (1)
$U_{2}$	U-value using formula (2)
$U_{3}$	U-value using formula (3)
$U_{c}$	average U-value of *U*${}_{1}$, *U*${}_{2}$, and *U*${}_{3}$
ϵ	Wall emissivity
σ	Stephen–Boltzmann constant: $5.6703\times 10^{-8}\;W\times m^{-2}K^{-4}$
$T_{s}$	Surface temperature
$\alpha_{c}$	convective heat coefficient
$T_{ai}$	Constant
$T_{in}$	Internal wall temperature
$T_{out}$	External wall temperature
$T_{ref}$	Reflexive wall temperature
$c_{1}$	Convective heat coefficient constant
*L*	Height of the wall
ASHRAE	American Society of Heating, Refrigerating and Air-Conditioning Engineers (ASHRAE)
